# Seasonal and Long-Term Population Dynamics of the Peach Fruit Fly in Egypt

**DOI:** 10.3390/insects16040332

**Published:** 2025-03-21

**Authors:** Mustafa M. Soliman, Esmat A. EL-Solimany, Thomas Hesselberg, Amira A. K. H. Negm

**Affiliations:** 1 Entomology Department, Faculty of Science, Cairo University, Giza 12613, Egypt; 2 Plant Protection Research Institute, Agricultural Research Center (ARC), Giza 12619, Egypt; elsolimany2014@yahoo.com (E.A.E.-S.); amiranegm2000@gmail.com (A.A.K.H.N.); 3 Department for Continuing Education, University of Oxford, Oxford OX1 2JA, UK; thomas.hesselberg@biology.ox.ac.uk; 4 Department of Biology, University of Oxford, Oxford OX1 2JA, UK

**Keywords:** *Bactrocera zonata*, population dynamics, temporal trends, temperature effects, pest monitoring

## Abstract

The peach fruit fly (*Bactrocera zonata*), a significant polyphagous pest, poses a considerable threat to fruit crops across its expanding range. Although climate change significantly impacts pest populations, its effects on *B. zonata* remain understudied. This research examined *B. zonata* population dynamics across two distinct Egyptian ecological zones (Sohag and Ismailia Governorates) from 2013–2023 using pheromone traps and climate data. Results revealed significant spatial and temporal variations in abundance patterns. Both regions displayed a unimodal distribution, with Sohag exhibiting a distinct peak during September to November, whereas Ismailia showed a broader peak period spanning from August to December. Temperature significantly influenced population levels while precipitation showed no significant effect. Similarly, our results indicated increasing population trends in both regions despite no significant long-term temperature changes. These findings suggest that factors beyond temperature alone, such as host fruit availability, regional environmental variations, and potentially evolving agricultural practices, drive *B. zonata* population growth, highlighting the need for comprehensive, climate-responsive pest management strategies that account for regional variations.

## Simple Summary

The peach fruit fly is a major pest that harms fruit crops. This study looked at how its populations changed over a 10-year period (2013–2023) in two different regions of Egypt. This study aimed to understand how climate affects these flies, as this information is important for protecting fruit crops. The researchers used special traps to count the flies and collected weather data in both regions. The findings showed that the number of flies peaked at different times in each region. In Sohag, fly numbers were highest from September to November, while in Ismailia, high numbers lasted longer, from August to December. Temperature played a big role in fly numbers, but rainfall did not. Interestingly, the fly population grew in both regions over time, even though temperatures remained stable. This suggests that other factors besides temperature are important for the flies’ growth. The study emphasizes the need for better pest control strategies that take into account both local conditions and climate changes to protect fruit crops effectively.

## 1. Introduction

Climate change is a global phenomenon with far-reaching consequences for agriculture and pest dynamics. It is characterized by rising global average temperatures, shifts in rainfall patterns, and an increase in extreme climatic events. These changes directly impact insect pest populations by affecting their developmental rates, voltinism, dispersal, and survival. Temperature, as a primary driver, plays a crucial role in insect population dynamics [1]. Studies have demonstrated that elevated temperatures can influence pest behavior and abundance [1,2,3,4], leading to potential range expansions and increased pest pressure in various regions. Climate change necessitates adapting pest management strategies to account for altered pest distribution, behavior, and population dynamics [1,4]. Effective pest management requires robust monitoring programs to track pest population dynamics and assess the effectiveness of control measures. Long-term population studies are essential for understanding pest responses to environmental changes, including those driven by climate change.

Egypt, with its diverse climatic and agricultural zones, presents a unique setting for examining the effects of climate change on pest dynamics. The country’s northern regions typically experience a Mediterranean climate, while the southern regions are characterized by a more arid, desert climate. These contrasting climatic conditions directly influence agricultural practices and the prevalence of various pests, including the invasive peach fruit fly (*Bactrocera zonata*) [5]. Furthermore, these regions offer valuable insights into how climatic variations impact peach fruit fly populations. By comparing peach fruit fly abundance and its relationship with climatic factors in these regions, this study aims to shed light on the temporal and spatial dynamics of this pest under the influence of climate change.

The peach fruit fly, *Bactrocera zonata* (Saunders), is a highly polyphagous pest, attacking over 50 host plants, including peach, guava, mango, apricot, fig, and citrus [6,7,8]. Native to South and Southeast Asia [6,9], *B. zonata* has invaded more than 20 countries outside its native range, including those in the Arabian Peninsula and Africa, posing a serious challenge to fruit production [6,9]. The economic impact of this pest is considerable, especially in Egypt, where it poses a threat to essential fruit crops. Infestations can result in yield reductions of up to 30–50% in severely affected peach and mango orchards [10,11]. The financial burden of pest management, which includes the use of pheromone traps and insecticides, further strains the resources of farmers. Additionally, stringent quarantine measures enforced by trading partners to prevent the spread of the peach fruit fly can limit Egypt’s access to international fruit markets, thereby exacerbating economic losses. In Egypt, *B. zonata* was first detected in 1997 and has become a dominant fruit pest, even surpassing the Mediterranean fruit fly in some regions, like El-Beheira Governorate [8,10,11]. This shift in dominance has been particularly evident in citrus orchards, where *B. zonata* now accounts for 65–75% of all fruit fly captures compared to only 20–25% for *Ceratitis capitata*, a complete reversal from the situation observed in the early 2000s [12]. This competitive displacement appears to be driven by several factors: the peach fruit fly’s higher reproductive potential (producing up to 400–500 eggs per female compared to 300–350 for *C. capitata*), its greater tolerance to higher temperatures (thriving at 30–35 °C while *C. capitata* populations decline above 32 °C), and its ability to infest fruits at earlier maturation stages [10,13].

Despite the well-documented impact of climate change on insect pests [1,2,3,14,15], there remains a limited understanding of its specific effects on *B. zonata* populations, particularly in Egypt [16]. Moreover, studies examining the population dynamics of *B. zonata* are relatively scarce, both globally [7,17] and within Egypt [8,10,11,12]. Existing research has primarily focused on the short-term effects of climate change on *B. zonata* population dynamics. This significant knowledge gap highlights the urgent need for comprehensive, long-term studies to better understand how climate change influences the abundance and distribution of *B. zonata* in Egypt. Here, we compare population dynamics between northern and southern regions of Egypt, evaluate relationships between climatic variables (temperature, precipitation, etc.) and *B. zonata* abundance, identify key factors influencing population changes in the *B. zonata*, as well as assess long-term trends in *B. zonata* abundance from 2013 to 2023.

## 2. Materials and Methods

### 2.1. Study Sites and Sampling of Peach Fruit Fly

**Figure 1:**
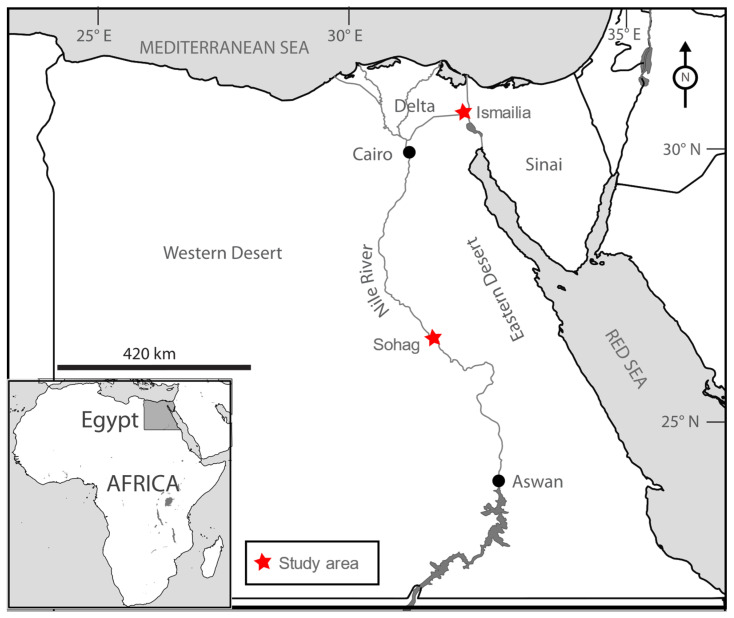
Map of Egypt showing the study localities.

This study was conducted in two distinct ecological zones in Egypt: one in the south, at the Shandaweel Agricultural Research Station Farms in Sohag Governorate (26°38′ N, 31°39′ E), and the other in the north, in Ismailia district, Ismailia Governorate (30°35′30″ N, 32°14′50″ E) (Figure 1). The Shandaweel site, managed by the Agricultural Research Center (ARC), encompasses over 225 acres of cultivated land. Its proximity to the Nile River and diverse vegetation, including permanent crops such as trees and orchards, as well as seasonal field crops and vegetables, makes it an ecologically significant area [18]. Between 2013 and 2023, four pheromone traps were installed in an area predominantly planted with citrus trees, with smaller adjacent areas containing mango, guava, and pomegranate. In contrast, the Ismailia district spans approximately 38,000 acres of cultivated land, of which 2758 acres are allocated to fruit crops. Mango and citrus are the dominant fruit crops in this region, supplemented by other crops such as olives, peaches, pears, apples, guava, palm, and fig [19]. From 2016 to 2018, a total of 214 pheromone traps were distributed among the fruit crops within this area.

At both locations, Jackson traps baited with sex attractants (methyl eugenol) were strategically placed approximately 300 m apart and positioned at a height of 1.5–2 m above the ground, ensuring effective trapping within the flight range of *B. zonata* [17]. Traps were monitored weekly to collect specimens and record data (Table S1). During each monitoring session, trapped specimens were collected, placed in labeled containers, and transported to the laboratory for identification using morphological keys specific to *B. zonata* [20,21]. Trap maintenance involved monthly cleaning of trap components and replacement of sticky liners, as well as replacing capsule lures every three weeks.

### 2.2. Climate Data Collection

Meteorological data, including mean monthly temperature, relative humidity, and total precipitation, were obtained from the NASA/POWER database (NASA Prediction of Worldwide Energy Resources) for both study sites in Sohag and Ismailia governorates spanning the period 2013–2023 (Table S2).

### 2.3. Data Analysis

Statistical analyses were performed to examine the relationships between *B. zonata* abundance and various environmental factors, as well as to detect temporal trends. Prior to model fitting, correlations between predictor variables were assessed to avoid multicollinearity. Relative humidity was excluded from the analysis due to its high correlation with the zone variable (r > 0.7). A generalized linear model (GLM) with negative binomial regression analysis (maximum likelihood estimation) was conducted using IBM SPSS Statistics Version 27 to analyze the relationship between *B. zonata* abundance and environmental factors. The negative binomial distribution was selected to account for overdispersion in the count data, which violated the assumptions of the Poisson distribution [22]. Monthly *B. zonata* abundance served as the response variable, while predictor variables included temporal factors (month, year), spatial factors (zone), and climatic variables (average monthly temperature, monthly precipitation). The statistical significance of each predictor in the model was evaluated using the Wald chi-square test. This test determines whether the regression coefficient (B) for each predictor differs significantly from zero, thereby indicating whether the variable exerts a meaningful effect on the abundance of *B. zonata*. To detect significant long-term trends in *B. zonata* abundance and temperature over the study period (2013–2023), we applied the Mann–Kendall test, a non-parametric test commonly used for time-series trend analysis. This test assesses whether there is a monotonic upward or downward trend in a dataset without assuming normality or requiring linearity. It was used to determine the direction and significance of the trend. A positive Z value indicates an increasing trend, while a negative Z value indicates a decreasing trend. The Sen’s slope estimator was also calculated to quantify the rate of change in *B. zonata* abundance over time. The test was performed separately for each geographic zone to account for regional differences in population dynamics. The Mann–Kendall test was performed using the trend package in R statistical software (version 4.4.1).

## 3. Results

### 3.1. Seasonal Population Dynamics Across Regions

**Figure 2:**
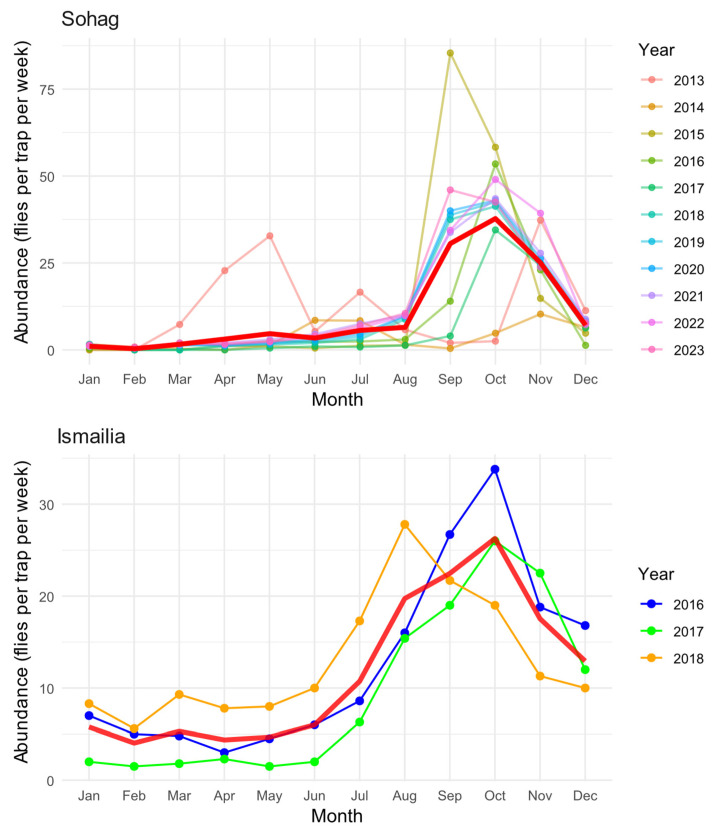
Seasonal patterns of *B. zonata* abundance in Sohag (2013–2023) and Ismailia (2016–2018) Governorates. Monthly abundance values are shown for each year (colored lines), with the overall average seasonal trend represented by the red line. Monthly values represent averages derived from four consecutive weekly trap catches. Abundance data were collected using pheromone traps (n = 4 in Sohag, n = 214 in Ismailia) and are expressed as mean catches per trap per week.

Our analysis revealed distinct seasonal patterns of *B. zonata* abundance across two Egyptian governorates, Sohag (2013–2023) and Ismailia (2016–2018) (Figure 2). In Sohag, the seasonal dynamics exhibited a predominantly unimodal pattern, characterized by a single prominent peak in abundance occurring during September and November, with maximum densities reaching approximately 85 flies per trap per week. A minor secondary peak was observed in May 2013, though this pattern was not consistent throughout other years. During the winter months (December–March), population levels remained consistently low, with fewer than 5 flies per trap per week.

The seasonal pattern in Ismailia also displayed a unimodal trend, albeit with a broader peak extending from August to December. The maximum abundance recorded in Ismailia reached approximately 35 flies per trap per week, representing less than half the peak density observed in Sohag. The population increase in Ismailia began gradually from July, reaching its peak in October, followed by a gradual decline through December. Winter and spring months (January–June) maintained relatively stable but low population levels, typically below 10 flies per trap per week.

Interannual variation was evident in both regions, particularly in peak abundance timing and magnitude. In Sohag, the years showed considerable variability in the September-November peak, ranging from approximately 40 to 85 flies per trap per week. Ismailia exhibited more consistent year-to-year patterns over the three years of observation, although 2016 displayed a notably higher October peak compared to 2017 and 2018. The mean seasonal trend (red line) effectively captures the overall pattern in both regions, smoothing out year-to-year variations while maintaining the characteristic seasonal dynamics specific to each location. This pattern suggests that while local climate and environmental conditions influence the timing and magnitude of population fluctuations, there is a consistent underlying seasonal rhythm in both regions.

### 3.2. Factors Influencing Peach Fruit Fly Abundance

Temporal patterns were strongly evident, with significant variation across both months (Table 1, Binomial GLM: χ^2^ = 298.1, df = 11, *p* < 0.001) and years (Table 1, Binomial GLM: χ^2^ = 41.4, df = 10, *p* < 0.001). Monthly patterns showed that *B. zonata* populations were significantly lower from January through August compared to December (reference month), with the strongest reductions observed in June (B [estimated regression coefficient] = −2.520, *p* = 0.003) and May (B = −2.356, *p* = 0.002). Population levels in September through November were not significantly different from December. Across the study period (2013–2023), notable fluctuations in abundance were observed. Compared to 2023 (reference year), significantly higher abundance was recorded in 2013 (B = 0.927, *p* = 0.003), while 2017 showed significantly lower abundance (B = −0.790, *p* = 0.009). Other years showed no significant differences from 2023, suggesting no consistent long-term trend in population changes. Spatial variation was pronounced between the two study regions (Table 1, Binomial GLM: χ^2^ = 24.6, df = 1, *p* < 0.001), with Sohag showing significantly lower *B. zonata* abundance compared to Ismailia (B = −1.110, *p* < 0.001). This geographical difference likely reflects the distinct climatic conditions between northern and southern Egypt. Regarding climatic variables, temperature showed a significant positive relationship with *B. zonata* abundance (Table 1, Binomial GLM: χ^2^ = 5.2, df = 1, *p* = 0.023), indicating that higher temperatures were associated with increased population levels. However, precipitation did not significantly influence *B. zonata* abundance (Table 1, Binomial GLM: χ^2^ = 0.2, df = 1, *p* = 0.655).

### 3.3. Long-Term Population Trends (2013–2023)

**Figure 3:**
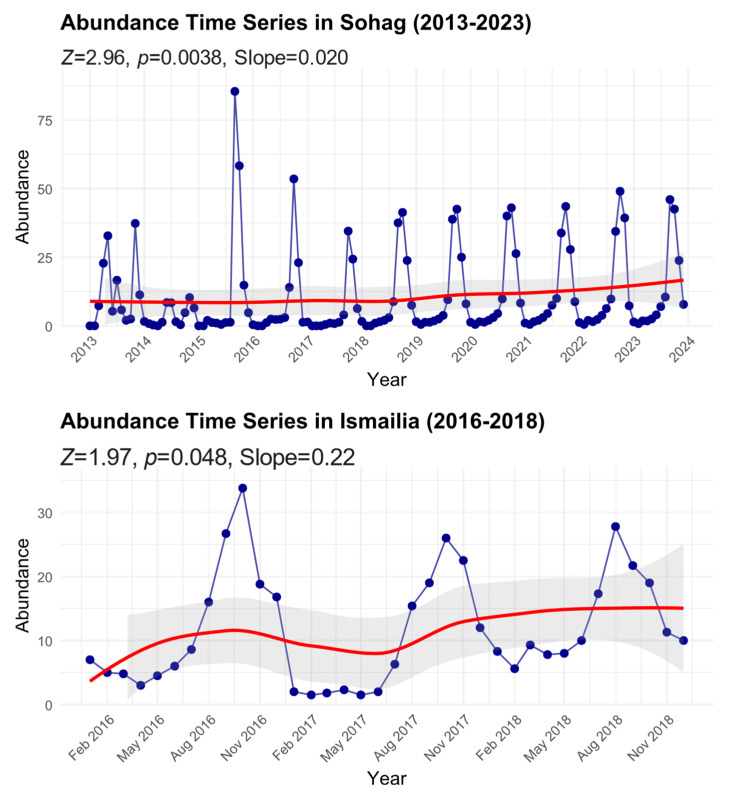
Time series of peach fruit fly abundance in Sohag and Ismailia Governorates from 2013 to 2023. Monthly data points with smoothed trend lines (red) are shown along with 95% confidence intervals (shaded area). Mann–Kendall trend analysis results, including the standardized test statistic (Z), significance level (*p*-value), and Sen’s slope, are reported for each time series to illustrate trends over the study period.

We found significant temporal increasing trends in *B. zonata* abundance across two Egyptian regions (Figure 3). In Sohag, where monitoring spanned a decade (2013–2023), the analysis showed a significant positive trend (Mann–Kendall test: Z = 2.96, *p* = 0.0038) with a Sen’s slope of 0.020, indicating a gradual but consistent increase in *B. zonata* abundance over the study period. The time series exhibited pronounced seasonal fluctuations, with peak abundance values reaching up to approximately 85 individuals during extreme events, particularly in 2016. Despite these periodic spikes, the smoothed trend line (red) demonstrates a steady upward trajectory, with the mean abundance increasing from about 8 individuals in 2013 to approximately 15 by 2023.

In Ismailia, despite the shorter monitoring period (2016–2018), the analysis also revealed a significant positive trend (Mann–Kendall test: Z = 1.97, *p* = 0.048) with a steeper Sen’s slope of 0.22, suggesting a more rapid increase in abundance compared to Sohag. The time series showed regular seasonal oscillations with peak abundance values reaching approximately 35 individuals. The smoothed trend line indicates an increase in the baseline abundance from about 5 individuals in early 2016 to roughly 15 by late 2018. The 95% confidence intervals (shaded areas) around the trend lines illustrate the degree of uncertainty in these temporal patterns, with wider intervals generally corresponding to periods of greater variability in the observations. This increasing trend in both regions, despite their different geographic and climatic conditions, suggests a broader pattern of growing *B. zonata* populations that may be linked to regional climate change or other environmental factors.

### 3.4. Temperature Patterns and Trends

**Figure 4:**
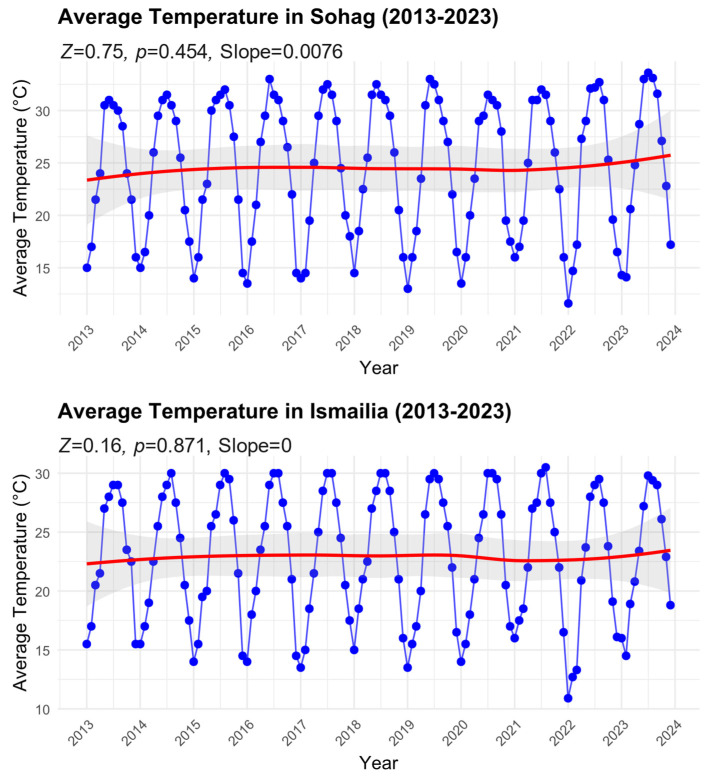
Time series of average temperature in Sohag and Ismailia Governorates from 2013 to 2023. Monthly data points with smoothed trend lines (red) are shown along with 95% confidence intervals (shaded area). Mann–Kendall trend analysis results, including the standardized test statistic (Z), significance level (*p*-value), and Sen’s slope, are reported for each time series to illustrate trends over the study period.

We found no significant temporal patterns of average temperatures for either region over the study period (Figure 4). In Sohag, despite a slight positive slope (Sen’s slope = 0.0076), the trend was not statistically significant (Mann–Kendall test: Z = 0.75, *p* = 0.454). The temperature time series showed consistent seasonal oscillations, with summer peaks reaching approximately 32 °C and winter troughs dropping to about 14 °C. The smoothed trend line (red) indicates a marginal increase in average temperature from approximately 23 °C in 2013 to 25 °C by 2023, though this change falls within the natural variability as shown by the 95% confidence intervals (shaded area).

Similarly, in Ismailia, the analysis showed no significant trend in temperature over the decade (Mann–Kendall test: Z = 0.16, *p* = 0.871, Sen’s slope = 0). The seasonal temperature pattern was comparable to Sohag but with slightly lower amplitude, ranging from around 11 °C in winter to 30 °C in summer. The smoothed trend line remained relatively stable around 22–23 °C throughout the study period, with minimal variation in the long-term average. The absence of significant temperature trends in both regions, despite the observed increases in *B. zonata* abundance (as shown in Figure 3), suggests that factors other than long-term temperature changes may be driving the observed increases in pest populations. The consistent seasonal temperature patterns in both regions, however, may help explain the regular seasonal fluctuations in *B. zonata* abundance.

## 4. Discussion

This study investigated the population dynamics of the peach fruit fly (*Bactrocera zonata*) across two distinct ecological zones in Egypt—Sohag in the south and Ismailia in the north—over a decade (2013–2023). A key finding is the shared seasonal rhythm observed in both regions. Despite some regional variations, *B. zonata* populations in both Sohag and Ismailia exhibited a unimodal seasonal pattern, characterized by a distinct peak in abundance and a period of low activity during cooler months. This fundamental similarity suggests common underlying drivers shaping their population dynamics across Egypt. However, within this shared framework, we also observed notable spatial and temporal variations. Specifically, while both regions showed a unimodal pattern, Sohag exhibited a more sharply defined peak occurring primarily between September and November, whereas Ismailia displayed a broader peak period extending from August to December. Temporal trends further revealed significant year-to-year and seasonal fluctuations in both locations, with populations in both regions positively associated with temperature and exhibiting increasing long-term trends despite stable temperatures.

*B. zonata* fly is active almost all year round in Egypt, except for short periods during the cold winter months [8,12]. Both Sohag and Ismailia exhibit seasonal patterns in *B. zonata* abundance closely tied to temperature trends. Fly activity is significantly reduced during the cooler winter months (January to March) when average temperatures drop below 20 °C, aligning with seasonal patterns of low abundance observed in both Sohag and Ismailia. However, some activity persists in regions like Ismailia at temperatures as low as 11 °C, suggesting that the developmental threshold for *B. zonata* is likely closer to 10–15 °C, consistent with prior studies [5,6]. Population surges align with rising temperatures in late spring and peak during the warmer months (July to October), underscoring the critical role of temperature in regulating the life cycle of *B. zonata*. Despite regional differences, both locations show an underlying seasonal rhythm, as captured by the mean seasonal trend, reflecting the influence of shared climatic drivers, particularly temperature, in structuring annual population dynamics.

Child [23] highlighted that ectothermic organisms, including insects, depend on external heat sources and nutrients for energy to sustain life and reproduce. Consequently, the variations in *B. zonata* abundance observed during the study period can be attributed to the surrounding temperature, which serves as the primary physical factor limiting insect distribution. Both this study and prior literature highlight temperature as a key driver of population dynamics. For instance, Sharkia data revealed population peaks associated with temperatures around 31 °C [8], consistent with our observations of high abundance during warm months in Sohag and Ismailia. Elevated temperatures are known to accelerate the development of immature stages of *B. zonata*, thereby contributing to rapid population growth [8]. This preference for warmer conditions likely explains the proliferation of *B. zonata* during Egypt’s summer months [10].

The pronounced temporal patterns, with significant variations across both months and years, highlight the dynamic nature of *B. zonata* populations. These fluctuations could be attributed to various factors, including seasonal changes in temperature, host plant availability, and natural enemy activity [6,11]. Similar trends were observed in global studies, where seasonality often dictated population peaks. For instance, the spatiotemporal study in Sargodha, Pakistan, reported peak activity during warm months (July–September), aligning with periods of host fruit availability and favorable temperatures [24]. Additionally, a study investigating the *B. zonata* distribution predicted that climate warming would amplify these seasonal fluctuations, especially in regions already experiencing moderate population levels [5].

Spatial differences were pronounced, with lower abundance in Sohag compared to Ismailia, reflecting climatic disparities between northern and southern Egypt. This mirrors findings in other regions, where geographical and climatic conditions were pivotal. For example, Sargodha’s citrus orchards exhibited variations due to microclimatic factors within orchards and between nearby regions [24]. Harris and Lee [25] noted that even within small geographic areas, microclimatic variations could profoundly influence fruit fly abundance, underscoring the importance of local conditions in shaping population trends. Sohag’s lower abundance could be attributed to its hotter and drier conditions, which may exceed the species’ optimal thresholds for survival and reproduction. Previous studies corroborate this observation, showing that extreme heat stress and aridity limit population growth [5,6]. In contrast, Ismailia’s milder climate supports greater abundance, aligning with the reported preference of *B. zonata* for subtropical environments with moderate temperature fluctuations. Furthermore, global suitability maps suggest that regions with moderate temperatures, like Ismailia, provide ideal conditions for *B. zonata* proliferation [6].

The significant positive relationship between temperature and *B. zonata* abundance confirms temperature as a key driver of population dynamics, consistent with general principles of insect ecology and previous studies on *B. zonata* [5,6,7,9]. These studies highlight *B. zonata*’s optimal development range between 25 and 30 °C and its limited survival below 12 °C or above 35 °C. The findings that higher temperatures are associated with increased *B. zonata* populations suggest that climate change could significantly impact the pest’s distribution and abundance. However, extreme temperatures can lead to mortality, as studies have shown that *B. zonata* populations decline during hot, dry seasons [8,12]. They seek refuge in humid, shady areas, feeding on honeydew produced by aphids infesting fruit trees [17]. Cold temperatures are also detrimental, as observed in India, where *B. zonata* remains inactive during the coldest months (December to mid-February) [17]. The absence of a significant effect of precipitation, however, suggests that temperature exerts a more dominant influence on population dynamics, particularly in arid and semi-arid regions such as Egypt, where precipitation is scarce throughout the year. Conversely, the high rainfall in subtropical areas supported host plant growth, indirectly boosting fly populations [5]. It is also possible that other factors, such as irrigation practices in agricultural systems, mitigate the effects of precipitation variability.

The relationship between mean abundance and population dynamics of the *B. zonata* differs markedly between the two study regions, requiring careful interpretation of the statistical analyses. The negative binomial regression indicates lower mean abundance in Sohag across the entire study period, while raw abundance data reveal higher peak populations in this region. This apparent contradiction can be explained by examining the temporal patterns in both locations. Population dynamics in Sohag are characterized by extreme fluctuations, with peak densities reaching 85 flies/trap/week during optimal conditions, followed by prolonged periods of very low abundance (<5 flies/trap/week). In contrast, Ismailia demonstrates more moderate but persistent populations, with peak densities of 35 flies/trap/week and maintaining baseline populations of approximately 10 flies/trap/week during off-peak periods. When averaged across the full study period, these patterns result in a lower mean abundance in Sohag despite its higher maximum values. This difference is likely influenced by environmental or ecological factors, including the availability and type of host crops. In Sohag, citrus—characterized by more concentrated fruiting periods—dominates as the primary host crop, leading to sharp but transient population peaks. Conversely, mangoes and citrus are the predominant crops in Ismailia, providing a more consistent host availability throughout the season, which supports stable populations and broader abundance peaks. These findings suggest the need for region-specific management strategies. In Sohag, control efforts should target the low-population periods during winter months (January–March), when flies are most vulnerable. This approach allows for precision interventions during population troughs, maximizing the efficiency of control measures. Managers should develop host-specific protocols aligned with citrus fruiting cycles, employing strategic baiting, trapping, and biological control methods during critical windows. Conversely, Ismailia’s more consistent population requires continuous, year-round monitoring and integrated pest management approaches focused on sustained population suppression.

The availability of suitable host fruits is pivotal in shaping the population dynamics of *B. zonata*. Population peaks are closely aligned with the fruiting seasons of key host plants, such as mango, peach, and citrus, as demonstrated in several studies [8,11]. For instance, Darwish [11] reported that *B. zonata* populations in Beheira Governorate reached their highest levels during the ripening period of peach orchards, underscoring the strong association between host fruit availability and fly abundance. Similarly, *B. zonata* populations in mango orchards exhibit growth from June to October, coinciding with the mango fruiting season, while citrus orchards experience population peaks from August to December, reflecting the citrus fruiting period [24]. The research findings reveal the critical relationship between population dynamics of the species and the temporal and spatial distribution of host fruits, which are essential for reproductive and developmental processes. This observation aligns with research conducted by Alberti et al. [26] in Brazil, which documented population fluctuations of 12 fruit fly species, including *Anastrepha fraterculus* and *A. grandis*, demonstrating strong correlations with host fruit availability in peach and passion fruit orchards. Our findings on seasonal population dynamics of *B. zonata* align with periods that typically coincide with host fruit availability in these regions, suggesting a potential relationship that warrants further investigation.

The decade-long monitoring in Sohag (2013–2023) revealed a gradual but consistent increase in *B. zonata* abundance. This aligns with global findings that climate warming contributes to gradual population increases in fruit fly species. For example, Martínez-Ferrer et al. [27] noted similar temporal increases in *C. capitata* populations in eastern Spain, driven by warmer winters and prolonged favorable conditions in spring and summer. In Ismailia, monitoring over a shorter period (2016–2018) revealed a steeper increase. Such rapid changes have also been observed in regions experiencing abrupt climate shifts or localized warming. Aluja et al. [28] emphasized that short-term monitoring often captures pronounced impacts of climatic anomalies, such as sudden increases in temperature or changes in precipitation patterns, on pest populations. The steeper slope in Ismailia might also reflect a region experiencing more pronounced environmental shifts, such as temperature variability or changes in agricultural practices. Furthermore, Ismailia’s proximity to the Nile Delta and associated stable microclimatic conditions could create an ideal environment for *B. zonata* proliferation compared to the hotter, arid climate of Sohag. The consistent upward trends in both regions, despite differing geographic and climatic conditions, suggest a broader pattern of increasing *B. zonata* populations influenced by regional climate change.

It is important to acknowledge that the observed increases in *B. zonata* populations likely result from a complex interplay of factors beyond just temperature. Interactions with other species within the ecosystem, including competitors, predators, and parasitoids, could significantly influence *B. zonata* dynamics. For example, the increase in *B. zonata* populations could lead to increased competition with other fruit fly species, such as the Mediterranean fruit fly (*C. capitata*), which shares similar host plants. In regions where both species coexist, the rise in *B. zonata* abundance could potentially displace *C. capitata*, as observed in some parts of Egypt where *B. zonata* has become the dominant fruit pest [8,9]. Dejene et al. [29] reported similar competitive dynamics in Ethiopia, where suppression of *B. dorsalis* led to a resurgence of *C. capitata*, suggesting that *B. dorsalis* may outcompete *C. capitata* in interspecific interactions, with the latter rebounding when the former is controlled. Furthermore, changes in *B. zonata* populations may affect natural enemy dynamics, including parasitoid wasps and predatory insects. Studies in Pakistan by Sarwar et al. [30] found that parasitism rates by *Diachasmimorpha longicaudata* on *B. zonata* varied significantly with seasonal abundance of the host, with decreasing efficiency during peak fly populations. This inverse relationship between pest density and natural enemy efficiency could partially explain the continued population growth we observed.

Changes in agricultural practices over the study period could also have contributed to the observed increases in *B. zonata* abundance. An expansion of fruit production areas could provide more resources for *B. zonata* populations to thrive. The intensification of agriculture, including increased irrigation and fertilizer use, might also enhance host plant quality and suitability for *B. zonata*. Conversely, changes in pesticide use patterns could have complex effects. While increased pesticide use might initially suppress *B. zonata* populations, it could also lead to the development of pesticide resistance, ultimately resulting in population resurgence. Additionally, shifts in crop rotation practices could alter the availability of host fruits throughout the year, influencing *B. zonata* population dynamics.

Comparing our findings to studies in other regions with similar climatic conditions provides further context. In parts of South and Southeast Asia, the native range of *B. zonata*, studies have documented similar seasonal patterns driven by temperature and host availability (e.g., Amin et al. [17] in Bangladesh; Choudhary et al. [7] in India). However, the specific magnitude and timing of population peaks can vary considerably depending on local conditions. For example, studies in subtropical regions of Pakistan and India have shown that *B. zonata* populations can thrive under a wider range of temperature and humidity conditions than observed in our study areas in Egypt [9]. This variation is also present in regions with Mediterranean climate, as in this study. In the Mediterranean basin, studies from Spain [27] showed increases in *C. capitata*, attributed to warmer temperatures.

## 5. Conclusions

This study contributes to the understanding of climate–pest relationships, focusing on the *B. zonata* in Egypt. Comparative analyses between the distinct climatic zones of northern and southern Egypt provide valuable insights into regional variations in pest responses to climate change. The findings have practical implications for enhancing pest management strategies under changing climatic conditions. Long-term monitoring programs, like the one implemented here, are critical for predicting pest dynamics and supporting sustainable agriculture. Future research should expand to broader geographic regions and incorporate additional environmental factors, such as soil moisture, land use patterns, and irrigation practices, to improve predictive models and develop targeted pest management strategies.

## Figures and Tables

**Table 1:** Negative binomial regression analysis (maximum likelihood estimation (MLE)) of *B. zonata* abundance in relation to temporal (month, year), spatial (zone), and climatic variables (monthly temperature, monthly precipitation). χ^2^ = Wald chi-square (model effects test); B = estimated regression coefficient; *p* = *p*-value. Significance codes: *** = *p* < 0.001, ** = *p* < 0.01, * = *p* < 0.05. Note: “Ref” indicates the reference category for categorical variables in the regression model. Reference categories are as follows: month = December, year = 2023, zone = Ismailia.

Factor	χ^2^	df	*p*	B	95% CI	*p*
**Month**	298.1	11	<0.001 ***			
Jan				−1.312	−2.0–−0.60	<0.001 ***
Feb				−2.024	−2.7–−1.3	<0.001 ***
Mar				−1.904	−2.6–−1.1	<0.001 ***
Apr				−2.158	−3.1–−1.1	<0.001 ***
May				−2.356	−3.8–−0.87	0.002 **
Jun				−2.520	−4.2–−0.86	0.003 **
Jul				−2.166	−3.8–−0.46	0.012 *
Aug				−1.780	−3.4–−0.10	0.037 *
Sep				−0.228	−1.7–1.3	0.765
Oct				0.431	−0.71–1.6	0.459
Nov				0.588	−0.10–1.3	0.096
Dec				Ref	–	–
**Year**	41.4	10	<0.001 ***			
2013				0.927	0.32–1.5	0.003 **
2014				−0.522	−1.2–0.14	0.127
2015				−0.048	−0.65–0.55	0.877
2016				−0.453	−1.0–0.11	0.120
2017				−0.790	−1.4–−0.19	0.009 **
2018				−0.142	−0.70–0.41	0.619
2019				−0.022	−0.63–0.59	0.944
2020				0.025	−0.59–0.64	0.938
2021				0.118	−0.48–0.72	0.702
2022				0.140	−0.47–0.75	0.656
2023				Ref	–	–
**Zone**	24.6	1	<0.001 ***			
Sohag				−1.110	−1.5–−0.67	<0.001 ***
Ismailia				Ref	–	–
**Temperature**	5.17	1	0.023 *	0.124	0.01–0.23	0.023 *
**Precipitation**	0.199	1	0.655	0.017	−0.06–0.09	0.655

## Data Availability

The original contributions presented in this study are included in the article/Supplementary Materials. Further inquiries can be directed to the corresponding author.

## Supplementary Materials

The following supporting information can be downloaded at: https://www.mdpi.com/article/10.3390/insects16040332/s1, Table S1: Weekly trap counts of peach fruit fly in Sohag (n = 4 traps; 11-year period) and Ismailia (n = 214 traps; 3-year period) Governorates. Table S2: Climate variables measured at the investigated localities in Sohag and Ismailia Governorates (2013–2023).
